# Coach leadership in a crisis context: Investigating effective coach behaviors during the COVID-19 pandemic with a process view

**DOI:** 10.3389/fpsyg.2022.1061509

**Published:** 2022-12-05

**Authors:** Chen Zhao, Sophia Jowett

**Affiliations:** School of Sport, Exercise and Health Sciences, Loughborough University, Leicestershire, United Kingdom

**Keywords:** online training, team psychological safety, Q-method, crisis leadership, coaching behavior, leadership context

## Abstract

**Introduction:**

Drawing from the crisis leadership conceptualization, this study aims to investigate coaches’ opinion patterns on effective leadership behaviors during the COVID-19 pandemic. The study used a process view to explore how coaches as leaders act in pre, during, and post-crisis phases.

**Method:**

Thirty-two fulltime professional coaches (28 males and 4 females) from individual and team sports who experienced the entire COVID-19 pandemic from January 2020 to July 2021 in the United Kingdom were invited to express their perceptions of effective leadership behaviors. The study used Q methodology to analyze coaches’ perceptions and experiences.

**Result:**

The study revealed that the most effective coach leadership behaviors occurred during-crisis phase, which has the most positive ratings (*n* = 48) compared to the pre-and post-crisis phases (*n* = 18). The study’s main findings highlighted different phases of the COVID-19 pandemic demand various effective countermeasures from coaches. These practical and successful experiences were summarized as: division of labor, athlete-centered, team-driven, consulting, safe environment, and online coaching.

**Discussion:**

The findings of this study further highlight (1) the importance of coach leadership in creating a safe environment as it provides a much better platform to prepare for a pre-crisis stage, (2) that coaches should employ more positive than negative behaviors while interacting with team members more frequently especially during the crisis period, reducing athletes’ negative feelings such as anxiety and worry, and (3) that the online training-related activities and interactions during the crisis time can be expanded to noncrisis times, as a crisis event can have positive implications for the future if handled properly.

## Introduction

Leadership behaviors are determined and shaped by context (Vella et al., 2010; Oc, 2018; Stoker et al., 2019), the context “has frequently been shown to influence the observed range or base rates of the leadership variables of interest, to change the nature of examined relationships and to threaten the generalizability of findings” (Oc, 2018, p. 230). The recent COVID-19 pandemic has drawn attention to examining leadership in the context of crisis (Wu et al., 2021; Garretsen et al., 2022). The COVID-19 pandemic has impacted individuals and organizations globally and has affected the sports industry. Various sports practitioners had to change their routines and lifestyles significantly (Taku and Arai, 2020; Bratland-Sanda et al., 2021). Moreover, major international sports events (e.g., 2020 Olympics games) had to be ceased and postponed (BBC Sport, 2020). The amount of research during the COVID-19 pandemic in sports covered sports populations such as athletes (Roberts et al., 2020), people with disabilities (Kamyuka et al., 2020), and personal trainers (Bratland-Sanda et al., 2020). However, coaches, as critical sports participants, received less research attention.

In sports, coaches as leaders play an important leading role within teams, squads, groups of athletes, and other practitioners (Arthur and Bastardoz, 2020; Cotterill and Fransen, 2021; López de Subijana et al., 2021), as they take responsibility for creating a safe social environment and conducting effective coaching activities (Fransen et al., 2020; Gosai et al., 2021). The pandemic has brought more challenges for coach leadership. The various quarantine and lockdown policies had subjected coaches and other sports practitioners to restrictions. Such restrictions directly affected athletes’ training time, with athletes’ average weekly training time decreased by 27.6% after the start of lockdown (Zinner et al., 2020). Also, athletes cannot return to the field effectively due to training at home, where the injury rate in the first game has risen sharply (Seshadri et al., 2021). At the same time, home confinement pushed coaches to seek new training routines to transform difficulties into opportunities, such as online training (Moreno-Tenas et al., 2021). Thus, it is meaningful to investigate how coach leaders overcame these difficulties and gained an understanding and experience from having to manage a global crisis.

The present study builds on recent research that aimed to explore crisis leadership as a process (Bundy et al., 2017; Wu et al., 2021) with the aim to investigate coaches’ leadership behaviors around the time of the COVID-19 pandemic. Accordingly, taking a process-oriented view, we examined coach leadership in multiple primary phases: pre, during and post-crisis phases. Viewing a crisis as a process addresses a novel question that can help coaches assign appropriate measures at specific times rather than utilizing particular characteristics of the coach throughout the crisis event (Wu et al., 2021). Such process view of crisis leadership also aligned with sports scholars’ view of the COVID-19 pandemic (Samuel et al., 2020). Samuel et al. (2020) conceptualized the COVID-19 pandemic as a “change event” with four distinct stages–“pre-COVID-19 pandemic stage,” COVID-19 pandemic stage A, B (during the crisis time), and C (return to normal time). The UK’s lockdown policies provided ideal time period divisions. The UK had a relative sufficient pre-crisis stage since it was not among the first countries with an outbreak for a while. According to the official information provided by the UK government, the phases of this pandemic were as follows: the pre-COVID-19 (before first national-lockdown, March 2020), During COVID-19, and post-COVID-19 (after July 2021) (GOV.UK, 2021).

Wu et al. (2021) have conceptualized crisis events and crisis leadership, and the discussion that follows elaborates on these conceptualizations while applying them to coach leadership. The crisis event has been conceptualized on the basis of three key characteristics: (1) “*Unexpectedness*,” crisis event is different from normal events that occur frequently, a crisis is a situation that would not provide too much preparation time to organizational leaders. (2) “*Salience*,” such salience mainly reflects the perceived significance of the impact and urgency of the response. With these two characteristics in mind, coaches would face and experience a crisis with little time for preparation while subjectively evaluating objective crisis events. Meanwhile, time is paramount in this evaluation process. It is expected that different coach leadership behaviors result in various detections and appraisals of crisis events (c.f., Wu et al., 2021) and psychological factors, directly and indirectly, influence the coaches’ decisions, such as personal traits and leadership style (Kajtna and Barić, 2009). The last characteristics of crisis event is (3) “*Disruption*,” while crisis is a type of disruption, it has great potential for an organization (e.g., Bundy et al., 2017). In sports, many coaches and athletes faced big challenges and changes of lifestyle and lack of self-fulfillment during the pandemic (Taku and Arai, 2020). It has been reported that among others, athletes’ sleep pattern, training intensity, and eating habits changed significantly during the lockdown period (Pillay et al., 2020). Also, athletes perceived more stress compared to normal time (di Fronso et al., 2022). However, it is worth noting that crises can also be transformed into opportunities if handled properly (James et al., 2011). For example, rookie and injured athletes had more time to prepare and recover because of the COVID-19 pandemic crisis (Schinke et al., 2020; Taku and Arai, 2020).

Regarding the definition of coach crisis leadership, we referred to the general definition of crisis leadership as was put forward by Wu et al. (2021, p. 3) whereby crisis leadership is defined “as a process in which leaders act to prepare for the occurrence of unexpected crises, deal with the salient implications of crises, and grow from the disruptive experience of crises.” This definition was applied to crisis leadership as manifested by coaches over a period that included three states: preparation, confrontation, and growth. During this dynamic process, coaches first need to react and prepare for a crisis event; then, coaches identify appropriate behaviors and/or carry out specific measures during the crisis period; lastly, coaches gain experience and further develop as leaders from the crisis event. It transpires from such a conceptualization that the crisis is an unexpected event with salience as it depends on coaches’ subjective evaluation of an objective event. Coach leadership often focus on coaches’ behaviors (Bormann and Rowold, 2016; Arthur et al., 2017; Cotterill and Fransen, 2021). Wu et al. (2021)’s co-word analysis also revealed that leadership behaviors are the most published topic. Therefore, to address crisis leadership focusing on coaches *per se* and the topic of leadership behaviors, our research aims to investigate coaches’ opinion patterns on effective leadership behaviors during the COVID-19 pandemic. We hypothesize that the different phases of the COVID-19 pandemic demand sports coaches to vary their leadership roles and/or have various countermeasures. At a practical level, this study provides experience and reference for coaches to respond to potential crisis events in the future. Any event that meets the three elements of a crisis mentioned above can be categorized as a crisis event, not only limited to a global pandemic. Also, the current research expanded on sports population researched (Bratland-Sanda et al., 2021) by studying coaches’ leadership behaviors around the COVID-19 pandemic, and contributed to the literature during the COVID-19 pandemic in the realm of sports.

## Materials and methods

### Q methodology

**FIGURE 1:**
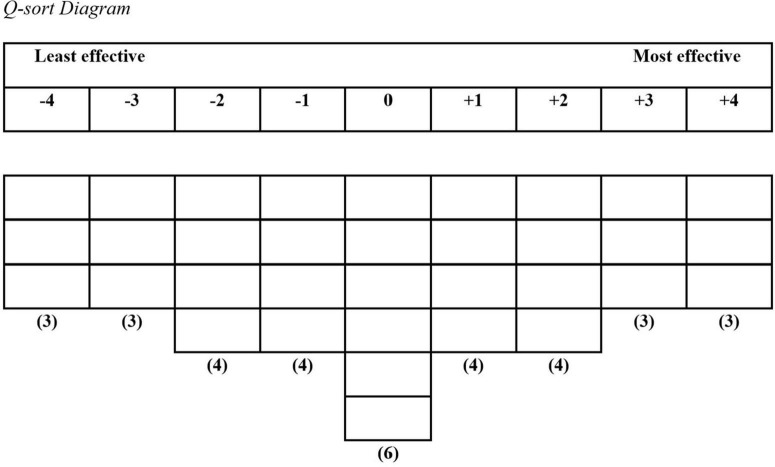
Q-sort diagram. Participants sorted statement (coaches’ behaviors) into the grid from (–4) for the least effective behaviors, (0) for the neutral behaviors, to (+4) for the most effective behaviors.

Since the current general definition of crisis leadership emphasizes leaders’ subjectivity to evaluate objective crisis events, we applied the Q methodology for data collection and analysis to examine coaches’ subjective viewpoints about coach leadership behaviors around the crisis time. Q methodology is a comprehensive approach for exploring human subjective viewpoints (McKeown and Thomas, 2013). One of the key characters is that Q-method emphasizes self-reference. As McKeown and Thomas (2013, p. 2) stated, “The primary purpose of undertaking a Q study is to discern people’s perceptions of their world from the vantage point of self-reference. These viewpoints constitute the Q methodological understanding of subjectivity.” The term subjectivity here is referred to “by which is meant simply an individual’s personal point of view on any matter of personal or social importance” (Wolf, 2010, p. 250). For the purpose of this study, we will examine how frontline coaches view effective coach leadership behaviors in a crisis event. Specifically, coaches will sort a set of statements (coach leadership behaviors during crisis time) on a bipolar scale from −4 to +4, as shown in Figure 1. Participants can sort statements (Q-sample) using paper cards or online software, namely Q-sorting. The Q-sample can be developed by existing public resources (e.g., coaches’ Tweets about their thoughts) or by in-person interviews with coaches. Our Q-sample was developed by interviewing because “In-person interviewing is most consistent with the principle of self-reference” (McKeown and Thomas, 2013, p. 3). Also, the statements generated from the interviews more realistically and accurately reflect how coaches respond to crises compared to inferring their behavior from other resources such as archived material (e.g., books and tweets). Last, our Q-sample was developed naturalistically (unstructured) as opposed to ready-made (structured) due to the current theory being underdeveloped (McKeown and Thomas, 2013). The validity of Q-method had been demonstrated in the content of sports (Chen, 1996; Harris, 2018), psychology (Watts and Stenner, 2014), and leadership (Podsakoff et al., 1990; Militello and Benham, 2010; Howard and Dhillon, 2021).

### Q samples and participants

The study followed the Code of Practice on Investigations Involving Human Participants issued by Loughborough University (2021). Data collection commenced once the study was granted approval by the University Ethics Approvals Sub-Committee of the authors’ institution. This study’s purpose was communicated directly with participating coaches, mainly through LinkedIn. All participating coaches voluntarily joined the study on their own time. The Q-sample was developed by interviewing 13 frontline professional coaches in the UK (female =1, male =12) from both team and individual sports (e.g., football, swimming, and volleyball). They all experienced the entire COVID-19 pandemic from January 2020 to July 2021. They shared how they managed and dealt with behaviorally and psychologically during the COVID-19 pandemic. All online conversations were audio recorded, with the coaches’ consent. The average interview length was 35 min, the maximum was 52 min, and the minimum duration was 25 min. All interviews were transcribed into 60 double-space pages of Microsoft Word files. Regarding the number of statements (Q samples), we were guided by Militello and Benham’s (2010; Q sample = 33) published research in *Leadership Quarterly* and Gabor and Cristache (2021; Q sample = 30) published research in *Mathematics*. A total of 34 statements were extracted and categorized as pre, during and post-crisis themes. The process was repeated by the first author to ensure consistency and avoid under-sampling or oversampling (see Table 1).

**TABLE 1:** Statements scores for each pattern.

		Patterns					
#	Q-sample (statements of Q-sorts)	1	2	3	4	5	6
1	Pre: Control the number of coaching team members (decision-makers) to be 3 or less	−2	−4	−3	−2	−4	0
2	Pre: Early acceptance of the significance of the crisis and start communicating with others regarding the crisis	0	−1	−2	3	−1	−3
3	Pre: Be transparent and clear when informing athletes about the crisis	3	−3	2	−1	−2	1
4	Pre: Always maintain a non-antagonistic and safe team culture	0	0	0	1	4	1
5	Pre: Reassure players we (the coaches) are still there, and we are still a team	3	2	1	0	−1	4
6	Pre: Prepare training program despite of compromises and limitations	2	3	0	−1	0	−2
7	Pre: Gather/update information about the COVID-19 pandemic from other sources before it hit the UK	−2	2	−2	0	−3	−2
8	Pre: See what other teams were doing, pause, and think	0	−1	−3	−1	−3	−1
9	Pre: Don’t panic, don’t rush, calm down, and discuss in meetings. It’s okay to have a break	1	−1	−2	3	3	−3
10	Pre: Clear assignment of responsibility to other team members (e.g., physio, captain)	3	0	0	−2	−3	−1
11	During: Be reactive instead of proactive	−3	−3	−4	−3	−2	−3
12	During: Organize team social activities to involve players (e.g., running group for charity, online quiz)	2	0	−1	−2	4	0
13	During: Consult professional advice and be strict about it	−1	−2	−1	2	−2	−4
14	During: Make sure athletes are able to access training resources all the time and not feel left out	1	1	4	4	3	2
15	During: Provide a suitable online platform for athletes to come and talk freely	0	2	0	0	1	3
16	During: Check on individual athletes regularly (e.g., phone call or message)	4	−2	2	2	2	4
17	During: Keep a positive and optimistic attitude and not transmit negativity to athletes	4	4	1	0	2	1
18	During: Keep reminding athletes that it’s their responsibility to maintain professionalism and to be ready to resume competing at moment notice	−2	−4	3	2	−4	−2
19	During: Physical training program was not to push athletes, focus on mental as much as physical	0	0	−4	4	1	0
20	During: Proactively communicate with athletes. If there are no updates, then tell athletes there are no updates, don’t say nothing	2	−2	4	4	2	0
21	During: Don’t expect much from the team	−4	−3	−4	−3	4	−4
22	During: Expand team training program to emphasize athletes/team development (e.g., invite seminar speakers)	−3	−1	2	1	−1	4
23	During: Be patient coping with crisis	1	1	1	−2	3	2
24	During: Discover hidden talents among athletes that could help other team members	−1	1	0	−3	0	−1
25	During: Design training program documents for athletes who cannot attend regular training sessions	0	0	4	1	−4	−1
26	During: Take up responsibility beyond usual coaching duties to better deal with the crisis	−1	−2	−3	0	1	1
27	During: Use feedback loop between coach and athlete to encourage coaching	−1	4	3	0	2	2
28	Post: Read guidelines from all relevant resources and plan how to come back	1	2	2	2	0	2
29	Post: Safe and guided return, not too much and too quickly	4	3	1	3	−1	0
30	Post: Restart normal intensity/competition as soon as possible	−4	−4	−1	−4	0	−4
31	Post: Continue to do effective new routines from crisis time (e.g., online session)	−4	1	−1	1	−2	3
32	Post: Offer different options of returning to training, let athletes choose how often they like to train	−3	4	−2	−4	0	−2
33	Post: Care more about how people are feeling and be emotional supporters	2	3	3	−1	1	3
34	Post: Ask others (e.g., athletes) to help manage	−2	0	0	−4	0	0

+4: most effective behaviors, −4: least effective behaviors, and 0: neutral behaviors.

An extensive person sample of 30–50 participants in the Q methodology is sufficient (McKeown and Thomas, 2013; Gabor and Cristache, 2021). Under the condition that the diversity of opinions is ensured, the Q method perfectly achieves the goal using a small number of samples (Militello and Benham, 2010; McKeown and Thomas, 2013; Gabor and Cristache, 2021). The selection of participants needs to be careful and to bring more subjectivity. A total of 32 full-time coaches (included interview phase coaches) from both individual and team sports (28 male and 4 female) who experienced the entire COVID-19 pandemic from January 2020 to July 2021 participated in the study. The age range was from 21 to 57 years old (SD = 10.16; mean = 31.34). The participants of this study represented sports included football (*n* = 11), rugby (*n* = 6), cricket (*n* = 3), swimming (*n* = 3), athletics (*n* = 3), volleyball (*n* = 2), basketball (*n* = 2), handball (*n* = 1), and table tennis (*n* = 1). About half of the participants (*n* = 15) coached at club level, the rest of the participants coached at international (*n* = 7), national (*n* = 7), and university (*n* = 3) level.

### Administering the Q sort

Due to the social restriction imposed by the COVID-19 pandemic, all sorting was administered through a “Q method software” (Wired Solutions, Windsor, ON, Canada) (Lutfallah and Buchanan, 2019). The software allows respondents to conveniently sort using a web browser. Coaches received a detailed introduction through text and video (2 min) by the software. First, coaches have a familiarization phase, in which they assembled and sorted the statements into three initial categories–most effective, neutral, and least effective behaviors before finalizing the sorting. Then, coaches sorted statements into the grid displayed in Figure 1 using (−4) for the least effective behaviors and (+4) for most effective behaviors. Thirty-two independent diagrams identical to Figure 1 were generated, representing each coach’ point of view. The average sorting time was 20 min, ranging from 18 to 22 min.

### Statistical analysis

Q methodology used centroid analysis to find correlation matrixes among the 32 Q-sorts, and Varimax rotation was applied to maximize the variance of the extracted patterns (Watts and Stenner, 2012; Gabor and Cristache, 2021). Six patterns with an eigenvalue greater than one (Watts and Stenner, 2012; Gabor and Cristache, 2021) were extracted and rotated, which explained a total of 49% of the study variance. All patterns’ composite fidelity meets the cut-off of 0.8 for explaining purpose (Gabor and Cristache, 2021). Each patterns implied a group of participants who shared similar opinions about the topic (Watts and Stenner, 2012). In our case, each pattern (or named factor) implied a group of coaches who had similar viewpoints about coach leadership behaviors around crisis time. Due to space constraints, we report the sorting value scores of each statement within each one of the three chronological patterns in Table 1 (value from −4 to +4) rather than attaching six sorting diagrams. Each pattern pre, during and post-crisis stages is explained next.

## Results

**FIGURE 2:**
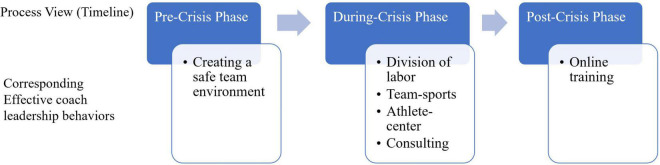
Six pattern of effective coach crisis leadership behaviors with a process view.

Overall, most of the coaches viewed behaviors in the during-crisis phase as the most effective behaviors. In contrast, pre-and post-crisis phase behaviors are either treated as less effective or not essential. Moreover, a close examinations indicated six patterns emerged describing the coach crisis leadership, namely, division of labor, athlete centered, team-driven, consulting, safe environment, and online training (see Figure 2). Each pattern represents a group of coaches with similar opinion about effective and ineffective behaviors taking place at pre, during, and post-crisis phases. To summarize and name each pattern, we identified extreme statements (e.g., ±4) that differed most from other patterns, extracted the “distinguishing statements” provided by Q software, and as well as coaches’ interview answers. An example of a distinguishing statement would be that statement 10 was only rated positive by the group of coaches represented by Pattern 1, whereas other groups of coaches rated statement 10 either negative or neutral (Table 1). Militello and Benham (2010) also suggested referencing other data (in our case, initial interview transcripts) would provide a rich data set to understand the area of crisis leadership research. We chose to interpret the most prominent and unique points of coaches’ view. The shared viewpoints, such as coaches should keep positive, would not be described every time. Following the reporting structure suggested by researchers (e.g., Watts and Stenner, 2012), the details of each pattern which represents similar opinions shared by a group of coaches, are presented below.

Pattern 1 referred to *division of labor*. This pattern had an eigenvalue of 7.65 and explained 24% of the study variance. Six male coaches from individual and team sports with an average age of 29 are significantly associated with this pattern. These coaches, affiliated with different coaching levels, strongly believed their coaching philosophy. As leaders, they had clear plans for team members. First, before the start of a crisis, they were the only group placing emphasis on assigning responsibilities to the team members (statements 10: +3). Coach #1 commented: “Because we cannot train athletes as we used to, we gave control over the program to the physio and S and C (strength and conditioning) coach. That’s one of the most effective behaviors we did. Both the physio and S and C coaches put some programs together so that athletes can come online and train in some way.” Focusing on how other teams cope or gather information about the pandemic was regarded as inefficient behaviors (statement 7: −2; statement 8: 0). Second, during a crisis, they were the only group of coaches who did not like to receive feedback from athletes to help their coaching (statement 27: −1) and rated such behavior ineffective. Finally, they resumed pre-pandemic training routines and were not in favor of applying the practices employed during the crisis times to post-pandemic times (statement 31: −4).

Pattern 2 referred to the notion of *athlete centered*. This pattern had an eigenvalue of 1.78 and explained 6% of the study variance. Two individual sports (triathlon and swimming) coaches with an average age of 53 were significantly associated with this pattern. They revealed an athlete-centered viewpoint to guide coaches’ behaviors. They coached at the club and international levels. In the pre-stage, they believed actions were more effective than psychologically accepting the severity of the crisis or using relaxed state of mind (statement 2, 9: −1; 6: +3). Specifically, they reassured the athletes that the coaches would get through this crisis with everyone (statement 5: +2). Also, they actively collected information and data about crisis events to prepare adequately for subsequent training (statement 7: +2; 6: +3). However, the transparency of crisis events is limited to the coaching staff, and letting athletes know too much about crisis events was considered ineffective leadership behavior (statement 3: −3). During the crisis stage, they chose not to put too much pressure on athletes. Such attitude is reflected in the fact that they neither checked the athletes’ status regularly nor consulted other professional opinions to manage the team with a strict regime in place (statement 16, 13: −2). Instead of putting themselves into multiple roles (statement 26: −2), they patiently discovered athletes’ abilities to help the team (statement 24: +1). In the post-stage, they also choose to return to regular training safely, but they gave athletes autonomy to decide how they preferred to return to regular practice (statement 32: +4), which is different from the views of all other coaches. As coach #2 commented: “The pandemic changed a number of rules, like how you engage with the athletes. In this kind of strange time, the real and positive behavior from my end is that when we set everything up, make things available for athletes to choose to come, not imposing them.”

Pattern 3 referred to *team driven*. This pattern had an eigenvalue of 1.92 and explained 6% of the study variance. Four team sports (2x football and 2x rugby) male coaches with an average age of 28 were significantly associated with this pattern. The results are in stark contrast to Pattern 2’s individual-sport coaches. From a team perspective, giving athletes flexible options to return to training is ineffective in their view (statement 32: −2). Their behaviors were team driven. Coaching a number of athletes at once make team-sports coaches very proactive, and reactive behaviors are unacceptable (statement 20: +4; 11: −4). Team driven coaches kept reminding athletes to maintain a high-performance status (statement 18: +3) and designed training plans involving everyone–not individualized training/coaching (statement 25: +4). As coach #3 commented: “As a team, we’re trying to reassure everybody will be back soon. This is only temporary. Please don’t lose sight of preparing for a season. The other teams who are also affected will also be working hard to be ready, so we can’t afford to take a rest period.” Last, team coaches focused on emotional support for athletes and have studied the return process in the post-crisis phase (statement 33: +3).

Pattern 4 referred to *consulting*. It had an eigenvalue of 1.50 and explained 6% of the study variance. Five coaches with an average age of 34 were significantly associated with this pattern. Three out of the five coaches coached at the international level and showed a lot of unique behaviors. Accepting the significance of a crisis event was an effective behavior (statement 2: +3) and having a relaxed mind (statement: 9: +3) was also different from other groups. They are the only coaching staff that consulted other professionals and strictly followed them (statement 13: +2). As coach #4 commented: “Club doctors and our head physio gave us some excellent education on its (COVID’s) seriousness and the impact it could have on all of our lives. We did what we needed to do to respect that and look after our players and our coaching staff.” They are also the only group of coaches who expressed a great deal of care about athletes’ mental as much as physical health (statement 19: +4).

Pattern 5 referred to the creation of a *safe environment*. It had an eigenvalue of 1.53 and explained 5% of the study variance. Three male coaches with an average age of 27 were significantly associated with this pattern. They were a group of coaches focused on building a safe team environment. Thus, they were the only group that considered the value of developing and maintaining a safe social environment (statement 4: +4) and organized team social activities as an effective behavior (statement 12: +4). As coach #5 commented: “If your teams were doing well, they tend to get on better. Our group had a good vibe, and we started having quizzes and just social nights. Or we do quizzes online, which was probably one of the most effective behaviors straight from the get-go.” Last, these team safety-oriented coaches also kept a relaxed mind (statement 9: +3) as an effective pre-crisis behavior which only showed in international-level coaches’ patterns (Pattern 4).

Pattern 6 referred to online coaching and had an eigenvalue of 1.17 and explained 5% of the study variance. Four younger coaches with an average age of 23 years were significantly associated with this pattern. They were the only group of coaches willing to expand training content (e.g., online session) used during the crisis event to post-crisis phase (statement 31: +3). As coach #6 commented “I think there’s no reason why it can’t be included. But don’t overdo it. I’ll put on an online session that maybe lasts 30 min. When I’ve got a different team this season that, changes things slightly because you have to get to know your players again. Have an online session where we talk about what we want to achieve.” This group of coaches also made conscious efforts to bond with their athletes since they were willing to build a suitable online communication platform (statement 15: +3). In addition, reading the safe guide and relevant resources would be an intelligent choice (statement 28: +2). Also, our results indicated that giving more emotional support was one of the effective behaviors (statement 33: +3). This might be because not everyone in the team is ready, such as the athletes got infected with COVID-19 suffered more than others.

## Discussion

The purpose of this study was to investigate frontline coaches’ perspectives on what constitutes effective leadership behaviors, and the patterns of opinions coaches held during the COVID-19 pandemic. Six patterns emerged from the Q-method analysis capturing coaches’ process views of the crisis event (pre, during, and post) on effective leadership behaviors, namely (1) division of labor, (2) athlete-centered, (3) team sport, (4) consulting, (5) safe environment, and (6) online coaching. The following sections explain the application of these patterns within the three chronological phases (pre, during, and post) and discuss how coaches can quickly and practically adapt to crises, minimize the disruption during a crisis event and gain useful experience after a crisis event (Wu et al., 2021).

First, examining coach crisis leadership in a process view can inspire coaches to understand how to prepare for future crisis events in an orderly and efficient fashion. From our interview and Q-sorting results, building and maintaining a safe team environment is the most effective behavior in the pre-crisis phase. Such results aligned with the recent research about the importance of coaches in creating a psychologically safe team (Fransen et al., 2020; Gosai et al., 2021). The unexpectedness of crisis events often does not allow coaches sufficient amount of time to adjust their behaviors and make decisions. Such a situation could be a major test for coaches to establish a safe team environment which will benefit team members to prepare for crisis events without panic. Coach leadership is an antecedent for athletes’ and sports practitioners’ psychological safety (Fransen et al., 2020). Coaches who were perceived with more coach leadership behaviors were more likely to make team members feel psychological safety (Gosai et al., 2021). In addition, coaches are one of the directly affected sports practitioners by crisis events, they also need to balance their team and life (Taku and Arai, 2020). Therefore, maintaining a united and non-antagonistic team would serve as the most reliable measure to help coaches minimize the fear of the crisis. To achieve a harmonious sports team environment, coaches were advised to build and sustain quality coach-athlete relationships (Jowett, 2017; Jowett and Slade, 2021). Coaches and athletes are two core sports practitioners; a quality coach-athlete relationship can help them stand together and increase team cohesion (Hampson and Jowett, 2014) and collective efficacy (Jowett and Chaundy, 2004).

Second, coaches mainly focus on minimizing or reducing the damage caused by crisis events in this phase. The biggest challenge identified by coaches was the lockdown policy which potentially made the team members lose their sports practitioners’ identity and hindered psychological connections (Henriksen et al., 2020; Schinke et al., 2020). When the coaches are in a vulnerable situation, they used their leadership attributes to assign and empower the corresponding professionals to help the team through the difficulties. Therefore, coaches assigned different tasks to team members and consulted other professionals, such as medical advisors. Under the remote training model, coaches could not maintain sufficient communication, which would increase conflicts between coaches and athletes (Wachsmuth et al., 2018). Thus, seeking outside expertise would be a wise choice. Coaches also recommend actively engaging with athletes, in which team-sports coaches designed and provided training resources for all athletes. Such result endorsed the research that group-based training increases athletes’ ability (Pedersen et al., 2021) and athletes coped better when provided training programs (Fox-Harding et al., 2021). Another effective measure for coaches with plans and are athlete-centered is mainly to focus on keeping positive and do not transmit the negativity to team members. Such behaviors can decrease the pressure on team members since athletes already suffer depression in this stage due to game opportunities being withdrawn and other concerns (Lambert et al., 2022). Coaches also are advised to encourage athletes to join social activities to increase physical activity at home, such as encouraging athletes to upload their plank challenge videos to the team’s social media group. This is an effective measure since sharing one’s fitness image on social media promotes fitness activities (Godefroy, 2020).

Last, the post-crisis phase mainly concerned maximizing the learning experience after the COVID-19 pandemic. Overall, coaches agreed that a quick return is inefficient. After just experiencing a crisis event, active performers need to adapt to the new environment and the corresponding changes (Samuel et al., 2020). The sixth patterns of coaches’ crisis leadership behaviors aligned with previous research findings about effective measures during the COVID-19 pandemic, such as online training (Moreno-Tenas et al., 2021). The sixth pattern revealed that coaches acknowledged the efficiency of online training during home confinement and continued to use the online training in their normal training program post-crisis. Such findings also demonstrated a crisis event can be transformed into an opportunity if one handled it properly (James et al., 2011; Taku and Arai, 2020). It is also worth noting that younger coaches (average of 23 years old) are more likely to embrace new training programs developed during the crisis compared to other coaches who choose to go back to previous training routines. Such results also warrant future research focusing on the differences and consequences of the age of coaches on crisis event handling. The disruption caused by the crisis event challenged coaches’ training routines and pushed coaches to adjust their coaching behaviors, but not every coach is willing to adapt the changes. The age of the sport’s practitioners can make a big difference psychologically during a crisis event. A study (Turner et al., 2021) demonstrated that senior athletes did not have significantly negative emotions during the COVID-19 pandemic even with a lack of matches. In contrast, the anxiety and depression of college students increased significantly due to absence of physical exercise (Xiang et al., 2020). Coaches, as the core member of the team, should maintain a healthy mental state to better influence their team members. Therefore, more research is encouraged to focus on the impact the age and experience of coaches on crisis events and the corresponding countermeasures and means.

### Limitation and recommendation

The current study used Q-method to examine the coaches’ views on effective leadership behaviors which aligned with the subjectivity in the definition of crisis leadership. It also needs to note that there are many other research agenda for coach crisis leadership. First, the coach crisis leadership can be studied in other context that meets the three criteria of crisis, a global pandemic is only one type of crisis. Also, the study sample mainly reflects the male coaches’ crisis leadership behaviors due to limited female coaches’ participants. The systematic discussion of crisis leadership has just begun, and more research and work need to be contributed to this field. For example, future research is recommended to discuss the connection between crisis leadership and established leadership theories. Wu et al. (2021) indicated the utility of transformational leadership in the context of crisis. Our result also endorsed such findings where coaches proactively support and interact with athletes during the crisis phase is consistent with the individualized consideration behavior of transformational leadership. Future research could further discuss the application of established leadership models and theories to crisis leadership. Second, the interview and Q-sorting data in this study were all from coaches. Future studies could also consider the views of athletes and other team members about perceived coach crisis leadership behaviors as a way to compare the extent to which the athlete and coach data and corresponding results align. While Q-method is an appropriate exploratory approach to investigate coach crisis leadership, future research is recommended to apply other conventional methods such as multivariate and regressive statistical inferential approaches (Flores et al., 2022; Teixeira et al., 2022) to further develop this topic. Last, researchers could observe the positive effects generated from the crisis event. For example, examine the difference and influence between coaches who continue to coach online and abandon online coaching. Spotting the positive effects of a crisis event is as important as dealing with a crisis event effectively.

## Conclusion

The current study revealed coaches’ effective leadership behaviors patterns around the time of crisis. With a process view, sports coaches can conduct various measures to deal with evolving demands of team members in the pre, during, and post-crisis time phases. Results from coaches’ view patterns revealed that building a psychologically safe environment is the most effective measure to confront a crisis event. Keeping a positive attitude and proactively interacting with other sports practitioners would minimize the damage during a crisis. Introducing new activities to regular training routines, such as online training, would maximize the learning experience from a crisis event. The applications of crisis coach leadership behaviors can apply to any event that meets the criteria of a crisis event. Given the critical role of coach leadership and the context of crisis, more research involving diverse participants such as athletes and other methodological approaches would further promote our knowledge of how coaches can handle potential crisis events.

## Data availability statement

The raw data supporting the conclusions of this article will be made available by the authors, without undue reservation.

## Ethics statement

The studies involving human participants were reviewed and approved by the Loughborough University Ethics Approvals Sub-Committee. The patients/participants provided their written informed consent to participate in this study.

## Author contributions

CZ contributed to the design, implementation, and analysis of the results. CZ and SJ contributed to wrote the manuscript and approved the submitted version.
